# Absence of long-term effects of reproduction on longevity in the mouse model

**DOI:** 10.1186/1477-7827-12-84

**Published:** 2014-08-25

**Authors:** Juan J Tarín, Vanessa Gómez-Piquer, Silvia García-Palomares, Miguel A García-Pérez, Antonio Cano

**Affiliations:** Department of Functional Biology and Physical Anthropology, Faculty of Biological Sciences, University of Valencia, Burjassot, Valencia, 46100 Spain; Department of Genetics, Faculty of Biological Sciences, University of Valencia, Burjassot, Valencia, 46100 Spain; Research Unit-INCLIVA, Hospital Clínico de Valencia, Valencia, 46010 Spain; Department of Pediatrics, Obstetrics and Gynecology, Faculty of Medicine, University of Valencia, Valencia, 46010 Spain; Service of Obstetrics and Gynecology, University Hospital Dr. Peset, Valencia, 46017 Spain

**Keywords:** Fertility, Gender gap, Life-history traits, Parental investment, Physiological costs of reproduction, Trade-offs, Survival times

## Abstract

**Background:**

Most human demographic data, particularly those on natural fertility populations, find no relationship or even a positive association between fertility and longevity. The present study aims to ascertain whether there is a trade-off between fertility and longevity in the mouse model.

**Methods:**

The study was focused on the first litter produced by 10- to 14-wk-old hybrid (C57BL/6JIco female X CBA/JIco male) mice. A single female/male per litter was individually housed with a male/female at the age of 25 and 52 wk, respectively, until the end of reproductive life in females or natural death in males under controlled housing conditions. Post-reproductive females and virgin mice were reared until natural death. Cox regression models with forward stepwise variable selection were fitted to examine the effect of several fertility variables on expectation of survival times.

**Results:**

Virgin females displayed higher life expectancy than virgin males. The relative risk of dying for a virgin male at a particular age was 2.116 [99% confidence interval: 1.317, 3.398] times that of a virgin female. No significant differences on expectation of survival times between virgin and mated females, and between virgin and mated males were found. Furthermore, total number of pups at weaning and total number of litters produced by a dam/stud, time interval between mating and last litter, time interval between litters, and age at last litter were not significant predictors of expectation of survival times in both mated females and mated males.

**Conclusions:**

Like in most human studies, the present study evidences no relationship between total number of offspring/litters produced by a dam/stud and expectation of survival times. Moreover, the present data are in agreement with the general phenomenon of a bias in life expectancy in favor of females.

## Background

It is acknowledged for long time that there are trade-offs between different life-history traits/components such as growth or reproduction and lifespan (for review, see Speakman [1]). Trade-offs may occur at functional levels ranging from cellular, tissue and individual (physiological) to ecology (e.g., increased risks of predation when individuals engage in mating or parental activities, or foraging while acquiring energy and nutrients for the reproduction event) and population/evolutionary (genetic) (cited by Plumel et al. [2]). For instance, current reproduction entails physiological costs that may reduce future reproductive success and/or survival of reproductive adults. But current reproductive effort may also involve trading-off the size against number of offspring (for review, see Speakman [1]); trading-off the beneficial effects of pleiotropic genes (genes that control for more than one phenotypic trait in an organism) on reproduction against their harmful effects on survival at later ages [3]; or trading-off, also in a antagonistic pleiotrophic manner, the beneficial effects on reproduction of the hypothalamic-pituitary-gonadal (HPG) axis early in life against its negative effects on cellular function and senescence at later ages when the HPG-axis becomes dysregulated [4]. However, most human demographic data, particularly those on natural fertility populations, find no relationship or even a positive association between fertility (total number of offspring born) and longevity (for a systematic review, see Hurt et al. [5]; for reviews, see Le Bourg [6] and Mitteldorf [7]). Negative impacts of childbearing on female longevity appear to be mostly associated to lower income groups in which general health is poor or pre-industrial groups that had not adequate access to health care (for references, see Ricklefts and Cadena [8]). Noteworthy, Ricklefts and Cadena [8] analyzed 18 species of mammals and 12 species of birds housed in favorable-condition zoo environments. They found that under these favorable zoo conditions, number of offspring produced up to a given age and age at first reproduction did not affect lifespan of females. This study, however, was criticized by other authors who claimed that zoo data are not ideal to investigate life-history trade-offs because of sample size and data quality issues [9].

Larke and Crews [10] suggested that the reason by which studies in women fail to find a trade-off between parity/fertility and longevity lies in the fact that the relationship between these traits is irrelevant. Instead, they proposed that it is the amount of “somatic reserve capacity” (reserves accumulated during earlier life) left over after reproduction that determines longevity. According to this hypothesis, greater somatic reserve capacity after reproduction may be obtained through greater cultural and less somatic parental investment (i.e., less physiological costs derived from the production, gestation, post-natal care, feeding and protection of young) into any one offspring. Of note, the concept of “somatic reserve capacity” is ambiguous and cannot be quantified or tested. In contrast, the “physiological costs of reproduction” can be measured and analyzed at least in small mammals such as the mouse (for review, see Speakman [1]).

According to Speakman [1], the physiological costs of reproduction can be either direct or indirect. The direct costs in turn can be divided into (i) those derived from satisfying the demands of pregnancy and lactation including energy, macronutrients (like protein), essential amino and fatty acids, vitamins, calcium and other micronutrients; and (ii) the physiological and anatomical modifications necessary to achieve these demands including growth of mammary tissue during late pregnancy, and increase in the sizes of both the liver and the pancreas, absorptive surface of the intestinal mucosa and length of the intestinal tract during lactation. Indirect costs can also be divided into 2 sub-types: (i) those related with optional compensatory adjustments in other components of physiological functioning to save energy to meet the direct physiological costs including reductions in thermoregulation, physical activity and immunocompetence; and (ii) obligatory costs that are an inevitable physiological consequence of the reproductive event, including hyperthermia, disruption of sleep patterns and bone loss. Note that Speakman [1] included oxidative stress as an obligatory consequential cost of reproduction but the degree to which oxidative stress affects reproductive costs remains under debate [2, 11–13].

The present study aims to ascertain whether there is a trade-off between fertility and longevity in mice, first mated at the same chronological age and kept under controlled housing conditions in the presence of a fertile male/female until the end of reproductive life in females or natural death in males.

## Methods

### Production of first-generation (F_1_) mice

Data analyzed in this study were extracted from a database generated in two previous studies designed to ascertain the long-term effects of maternal [14] and paternal [15] aging on offspring’s reproductive fitness and longevity. All the animal experiments performed in these studies were conducted in accordance with the National Research Council’s publication Guide for the Care and Use of Laboratory Animals [16]. In the present study only data from the first litter (n = 34) of 10- to 12-wk-old hybrid (C57BL/6JIco female X CBA/JIco male) females crossed with 12- to 14-wk-old hybrid males (i.e., the first litter produced by reproductively young mice) were entered into the statistical analysis. Females were individually housed with a randomly selected male. When females exhibited physical evidence of pregnancy (i.e., the presence of a distended abdomen), the male was removed from the cage and females allowed to give birth and breast-feed her pups until weaning. At the age of 21 days (at weaning), male and female F_1_ offspring were separated and housed in groups of 10 in a 35.5 × 23.5 × 18.5-cm plastic cage with a floor area of 510 cm^2^ (30 cm × 17 cm) and a vertical height of 18 cm. F_1_ mice were checked once per day, 7 days a week, until natural death to ensure accurate determinations of death dates. Mice were fed a standard laboratory diet and tap water ad libitum and were maintained on a 14 L:10D photoperiod (lights on at 0800 h) in a temperature controlled room at 21 to 23°C.

### Reproductive fitness of F_1_ mice

At the age of 25 wk, a single F_1_ female per litter (if available) was individually housed for the rest of its reproductive life with a randomly selected 12-wk-old hybrid male in a 26.5 × 20.5 × 13.5-cm plastic cage with a floor area of 352 cm^2^ (22 cm × 16 cm) and a vertical height of 14 cm. When the male reached the age of 45 wk, it was replaced by another 12-wk-old hybrid male in order to prevent any effect of male aging on female fertility. Cessation of female’s reproductive life was defined as the age at the last parturition following which no more offspring were born for 3 months. After cessation of the reproductive life, females were housed in groups of five under the same light:dark cycle and temperature conditions as their virgin siblings.

Because of space limitations in the Animal House, reproductive fitness of F_1_ males was tested when males reached the age of 52 wk (instead of 25 wk as in females). At this time, a single F_1_ male per litter (if available) was individually housed for the rest of its life with a randomly selected 10- to 12-wk-old hybrid female in a 26.5 × 20.5 × 13.5-cm plastic cage with a floor area of 352 cm^2^ (22 cm × 16 cm) and a vertical height of 14 cm. When the female was 35- to 40-wk old, it was replaced by another 10- to 12-wk-old female in order to prevent any effect of female aging on male fertility.

### Statistical analysis

Mixed-effects (some effects are random and some are fixed) designs of analysis of variance (ANOVA) were used for comparisons of means. Cox regression models with forward stepwise (Likelihood Ratio) variable selection were fitted to examine the effect of several fertility variables (covariates) on expectation of survival times of F_1_ mice. The potential correlation among siblings within a particular litter was controlled by introducing a random variable into the ANOVA models and a categorical covariate into the Cox regression models that clustered F_1_ mice on their respective mothers/fathers. In the descriptive analysis of data shown in Table 1, significance was defined as P ≤ 0.006 [α/9; i.e., α = 0.05 corrected for multiple (n = 9) hypothesis testing using the Bonferroni method]. In the analysis of Cox regression models, however, significance was changed to P ≤ 0.01 [α/5; i.e., α = 0.05 corrected for multiple (n = 5) hypothesis testing]. The statistical analysis was carried out using the IBM SPSS Statistics, Version 22 (© Copyright IBM corporation et al. 1989, 2013).

**Table 1:** **Fertility and longevity metric variables of F**_**1**_ **mice sorted by virginity status and gender**

Metric variables	Virginity status	Gender	
		Females ^a^	Males ^b^
*Age at death (wk)*	*Virgin*	99.2 ± 2.4^c^ (7, 141)^d^	85.2 ± 3.4^e^ (1, 133)
*Age at death (wk)*	*Mated*	88.3 ± 5.2 (35, 138)	104.0 ± 3.8 (61, 149)
*Total number of litters produced by a dam/stud*		8.9 ± 0.7 (2, 16)	10.5 ± 1.1 (1, 19)
*Total number of pups at birth produced by a dam/stud*		56.8 ± 5.2 (4, 92)	97.2 ± 10.7^f^ (1, 169)
*Total number of pups at weaning produced by a dam/stud*		52.4 ± 5.0 (3, 88)	92.2 ± 10.1^f^ (1, 162)
*Age at last litter (wk)*		51.9 ± 2.8 (31, 80)	92.2 ± 4.3^g^ (54, 130)
*Time interval between mating & last litter (wk)*		34.1 ± 2.8 (6, 55)	40.2 ± 4.3 (2, 78)
*Time interval between litters (wk)*		3.8 ± 0.1 (3, 5.4)	3.7 ± 0.2 (2, 5.6)
*Time interval between last litter & death (wk)*		29.2 ± 4.7 (0, 88)	11.8 ± 4.2^h^ (0, 94)

^**a**^Sample size: 103 virgin and 29 mated females.

^**b**^Sample size: 94 virgin and 25 mated males.

^c^Values are means ± standard errors of the means (SEMs).

^d^Minimum and maximum values.

^e-h^Value significantly different from the female group (^e^P ≤ 0.005; ^f^P ≤ 0.003; ^g^P ≤ 0.0005; ^h^P ≤ 0.001).

## Results

Table 1 shows fertility and longevity metric variables of F_1_ mice (n = 251) sorted by virginity status and gender. Age at death was lower (P ≤ 0.005) in virgin males compared with virgin females. In the mated group, no effect of gender on age at death, total number of litters produced by a dam/stud, time interval between mating and last litter, and time interval between litters was found. In contrast, mated males versus mated females displayed higher total number of pups at birth (P ≤ 0.003) and at weaning (P ≤ 0.003) produced by a dam/stud, and age at last litter (P ≤ 0.0005), but lower (P ≤ 0.001) time interval between last litter and death.

**Figure 1:**
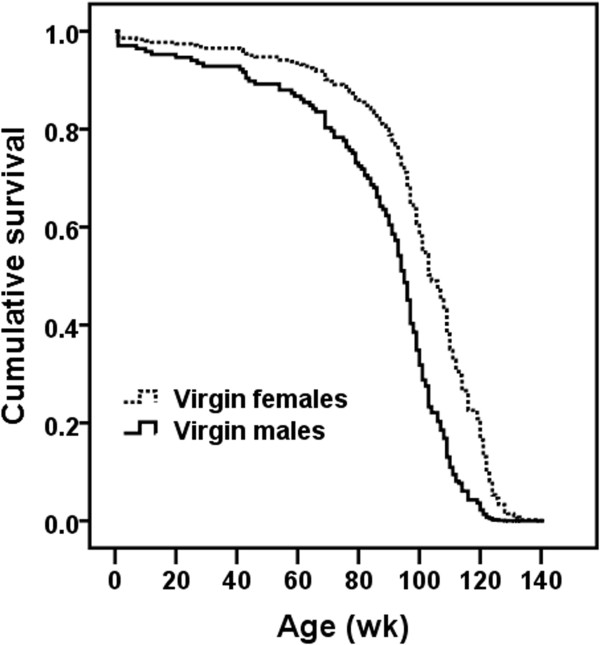
**Effect of gender on expectation of survival times in virgin mice.** Females displayed higher (P ≤ 0.0005) expectation of survival times than males.

**Figure 2:**
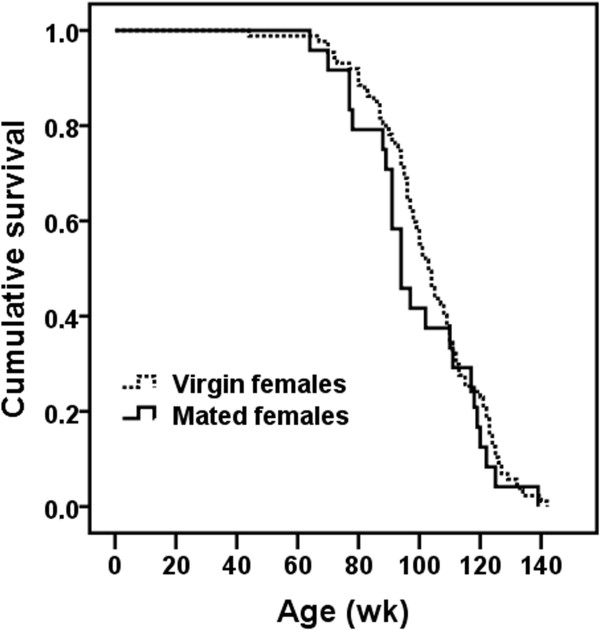
**Effect of virginity status on expectation of survival times in female mice.** No significant effect on expectation of survival times of virginity status was found.

**Figure 3:**
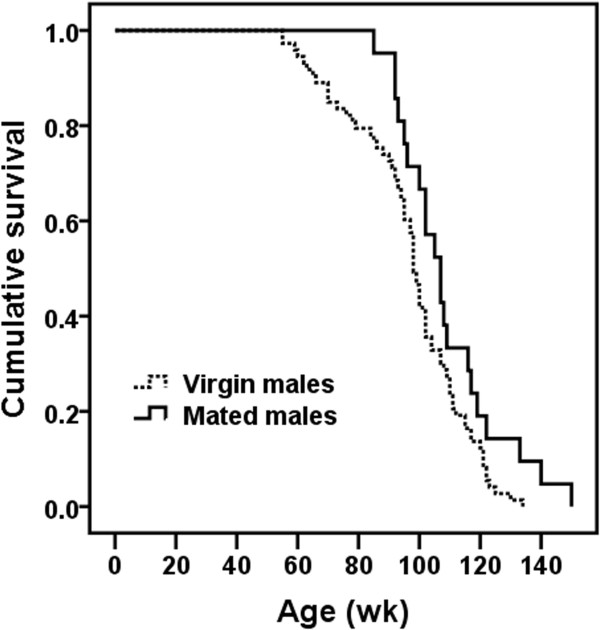
**Effect of virginity status on expectation of survival times in male mice.** No significant effect on expectation of survival times of virginity status was found.

A global Cox regression analysis testing for the effect on expectation of survival times of both gender and virginity status could not be performed due to differences between subgroups of mice in reproductive histories and recruitment regimens. In particular, all the virgin mice that entered into the study were recruited at birth without undergoing any selection process, i.e., their survival data were recorded from birth to death. On the contrary, mated mice were randomly selected among those virgin mice that were alive at the age of 25 (females) or 52 (males) wk, i.e., mated individuals were selected among those long-lived virgin mice (especially males). It is important to note that virgin and mated mice were reared in different density conditions. Although there are discrepancies among studies, in general, data suggest that increased density of mice induces a stress response, reduces immune function and increases aggressive behavior particularly in male mice (for review, see Whittaker et al. [17]). In addition, non-virgin males were mated just when most mated females ceased their reproductive life (at the age of 51.9 ± 2.8 wk; see Table 1). Consequently, further analyses were performed taking into account separately each gender and virginity status. In order to discriminate between short- and long-term effects of fertility on mortality and longevity, respectively, data from 5 mated females and 4 mated males (and their respective 13 and 6 virgin siblings) were excluded from the analyses because, by unknown reasons, they ceased reproduction before the age of 46 and 80 wk (the first quartile of the distribution of age at last litter in females and males, respectively), and died before or shortly after the age of 72.5 and 92.5 wk (the first quartile of the distribution of age at death in females and males, respectively; age at death: 35, 38, 41, 45 and 47 wk in females, and 61, 82, 93 and 95 wk in males). While we could not control for differences between virgin and mated mice in rearing conditions, in the Cox regression models applied, we controlled for differences in mating times between male and female mice.Accordingly, the first statistical analysis was focused only on virgin mice to ascertain the effect of gender on life expectancy. Survival data of females and males were compared from birth to death. Expectation of survival times of females (n = 103) was higher (P ≤ 0.0005) than that exhibited by males (n = 94) (Figure 1). The hazard ratio or relative risk, which can be interpreted as the predicted change in the hazard for a unit increase in the predictor, was 2.116 [99% confidence interval: 1.317, 3.398]. Note that females (the reference category) and males were coded as 0 and 1, respectively. Moreover, the hazard function is a measure of the potential for the event (in this case “to die”) to occur at a particular time t, given that the event did not yet occur. This means that the relative risk of dying for a virgin male at a particular age was 2.116 times that of a virgin female.The second and third statistical analyses were aimed to ascertain the effect of virginity status of females and males on expectation of survival times. Special care was taken to select only those virgin females that survived at least until the age of 25 wk and virgin males reaching the age of 52 wk (time at which mice were mated). No significant differences on expectation of survival times between virgin (n = 87) and mated (n = 24) females (Figure 2), and between virgin (n = 73) and mated (n = 21) males (Figure 3) were found.

The fourth Cox regression analysis should have been centered only on mated mice to ascertain the effect of gender on expectation of survival times. However, as mentioned above, survival curves of mated females and mated males cannot be compared due to gender differences in reproductive history and recruitment criteria. Accordingly, statistical analyses were performed taking into account separately each gender. In both mated females (n = 24) and mated males (n = 21), total number of pups at weaning and total number of litters produced by a dam/stud, time interval between mating and last litter, time interval between litters, and age at last litter were not significant predictors of expectation of survival times.

## Discussion

The present study evidences no effect of fertility on expectation of survival times in both male and female mice. This result is in line with most human demographic data reporting no relationship or even a positive association between fertility and longevity (for a systematic review, see Hurt et al. [5]; for reviews, see Le Bourg [6] and Mitteldorf [7]). Moreover, the present study shows that virgin female mice display higher life expectancy than virgin male mice. According to the Cox regression model applied, the relative risk of dying for a virgin male at a particular age was 2.116 times that of a virgin female. This result is in agreement with the general phenomenon observed in the animal kingdom of a gender gap or bias in life expectancy in favor of females (Gopalakrishnan et al. [18] and references therein; for reviews, see Smith [19] and Ginter and Simko [20]).

As mentioned above, mammalian female reproduction entails direct physiological costs including those satisfying the demands of pregnancy and lactation, and the physiological and anatomical modifications necessary to achieve these demands. In addition, there are also indirect physiological costs related with optional compensatory adjustments to save energy to meet the direct physiological costs, and obligatory costs that are an inevitable physiological consequence of the reproductive event (for review, see Speakman [1]). In this context, we should emphasize the fact that females use different strategies to offset particularly costly periods of reproduction in such a way that total energy requirements may not be greater than those needed by non-breading females. Moreover, repeated events of pregnancy/lactation have a protective effect on later life risks of osteoporotic bone fractures, which suggests that regrowth of bone is overcompensated in the post-weaning period (for review, see Speakman [1]). Thus, the physiological compensatory mechanisms used by females during/after pregnancy and lactation may neutralize the potential long-term effects of fertility on female life expectancy.

Noteworthy, Plumel et al. [2] have recently highlighted the potential involvement of other non-energy based mechanisms through which reproductive costs may reduce life expectancy of females. This proposal is based on the finding that female mice with reduced litters (litter sizes decreased by 2 offspring 2 days after parturition) exhibit higher levels of liver regucalcin (a.k.a. senescence marker protein-30) than females with enlarged litters (litter sizes increased by 2 offspring 2 days after parturition) and control females with unmodified litter sizes. We should bear in mind that regucalcin is a calcium-binding protein, whose amino acid sequence appears to be highly conserved among vertebrates, that plays an important role in organism aging and survival. Actually, aging rats and senescence-accelerated mice display reduced levels of liver regucalcin, and knockout mice lacking this protein have shortened lifespans (for references, see Plumel et al. [2]). Although this mechanism is appealing, we should bear in mind that the liver samples used in the proteomic analysis performed by Plumel et al. [2] were taken from female mice culled just after their pups were weaned on day 21 after birth, time at which lactating dams are exposed to high energy demands (for review, see Speakman [1]). As no other proteomic determinations were performed at later ages, it is not possible from these data to ascertain whether changes in levels of liver regucalcin were either permanent or transitory.

Similar to female mice, the present study evidences no relationship between total number of offspring/litters produced by a stud and expectation of survival times. This result is not surprising since the physiological costs of reproduction of laboratory male mice are minimal or negligible compared with females. On the contrary, males from polygynous or sexually promiscuous mammals including rhesus macaques exhibit increased mortality risks associated with dispersal, energy expenditure for mate attraction displays or intrasexual competition, fighting, and higher exposure to predation (Hoffman et al. [21] and references therein). Likewise, males from polygynous insects display decreased longevity due to costs of copulation and/or courtship (Papadopoulos et al. [22] and references therein).

## Conclusions

The present data are in agreement with the general phenomenon of a bias in life expectancy in favor of females. Moreover, like in most human studies, the present study evidences no relationship between total number of offspring/litters produced by a dam/stud and expectation of survival times. This conclusion is endorsed by the fact that mice were first mated at the same chronological age (25 wk in females and 52 wk in males) and reared under controlled housing conditions, with abundant food and high-quality care. Furthermore, laboratory female mice (unlike women) display a constant period of post-natal care, feeding and protection of young (≈21 days) which also affords to control for post-natal parental investment allotted to offspring. Obviously, this kind of study is difficult to be performed in human beings due to the wide range of time at first parturition, lactating period and socio-economic status found in every population.

## Abbreviations

ANOVA: Analysis of variance
F_1_: First generation
HPG: Hypothalamic-pituitary-gonadal.
